# Evaluation of the ΔV 4D CT ventilation calculation method using *in vivo* xenon CT ventilation data and comparison to other methods

**DOI:** 10.1120/jacmp.v17i2.5985

**Published:** 2016-03-08

**Authors:** Geoffrey G. Zhang, Kujtim Latifi, Kaifang Du, Joseph M. Reinhardt, Gary E. Christensen, Kai Ding, Vladimir Feygelman, Eduardo G. Moros

**Affiliations:** ^1^ Department of Radiation Oncology Moffitt Cancer Center Tampa FL; ^2^ Department of Human Oncology University of Wisconsin School of Medicine and Public Health Madison WI; ^3^ Department of Biomedical Engineering University of Iowa Iowa City IA; ^4^ Department of Radiation Oncology Johns Hopkins University Baltimore MD USA

**Keywords:** ventilation distribution, 4D CT, deformable image registration, evaluation, XeCT

## Abstract

Ventilation distribution calculation using 4D CT has shown promising potential in several clinical applications. This study evaluated the direct geometric ventilation calculation method, namely the ΔV method, with xenon‐enhanced CT (XeCT) ventilation data from four sheep, and compared it with two other published methods, the Jacobian and the Hounsfield unit (HU) methods. Spearman correlation coefficient (SCC) and Dice similarity coefficient (DSC) were used for the evaluation and comparison. The average SCC with one standard deviation was 0.44±0.13 with a range between 0.29 and 0.61 between the XeCT and DLV ventilation distributions. The average DSC value for lower 30% ventilation volumes between the XeCT and ΔV ventilation distributions was 0.55±0.07 with a range between 0.48 and 0.63. Ventilation difference introduced by deformable image registration errors improved with smoothing. In conclusion, ventilation distributions generated using ΔV‐4D CT and deformable image registration are in reasonably agreement with the *in vivo* XeCT measured ventilation distribution.

PACS number(s): 87.57.N‐, *87.57.nj*, 87.57.Q‐, 87.85.Pq

## I. INTRODUCTION

Pulmonary ventilation and perfusion are not uniform throughout the lung and this heterogeneity may be increased if pulmonary diseases are involved.^(1)^ Different imaging modalities are currently used clinically for pulmonary ventilation evaluation. Nuclear medicine modalities, including nuclear scintigraphy,^2^, ^3^ single photon emission computed tomography (SPECT),^4^, ^5^ and positron emission tomography (PET),^6^, ^7^ are most commonly used. Magnetic resonance imaging (MRI)^8^, ^9^ and computed tomography (CT)^10^, ^11^ are also capable of pulmonary functional imaging.

Recently, four‐dimensional (or respiratory gated) CT (4D CT) has been proposed for ventilation calculation using deformable image registration (DIR).^12^, ^13^, ^14^ In the so‐called HU algorithm, deformable image registration is applied between respiratory phases of a 4D CT image set. The deformation transformation calculated from the DIR process maps corresponding voxels between two different respiratory phases. The Hounsfield unit (HU) differences between the corresponding voxels of the two phases are used to generate the differences in local densities which are related to ventilation.^(12)^ Ventilation can also be calculated using the Jacobian of the deformation transformation^(13)^ or using the transformation directly (geometrically).^(14)^ Recently, promising results have been reported on the agreement between ventilation results obtained using the Jacobian method and xenon‐enhanced CT (XeCT) in a large animal model.^(15)^ The latter measures regional ventilation by observing the contrast gas, xenon (Xe), wash‐in or wash‐out rate on serial CT images and is considered a gold standard for regional ventilation imaging.^(16)^ Patient‐based comparative studies have also shown promising results between 4D CT vs. 4D‐PET using G68a‐labeled nanoparticles,^(17)^ and 4D CT vs. SPECT.^17^, ^18^ Ventilation data derived from 4D CT also demonstrated good reproducibility in both animal and human studies.^19^, ^20^, ^21^ This new ventilation calculation method using 4D CT has also been applied in lung disease detection,^(22)^ radiotherapy treatment planning studies,^(23)^ and assessment of radiotherapy response.^(24)^

The advantages of ventilation imaging using 4D CT include: 1) 4D CT is a mature, commercial and widespread technology; 2) no additional procedures, such as inhalation of a contrast, are needed, which makes the clinical implementation straightforward; 3) high spatial resolution of functional lung imaging can be achieved, which is one of the major advantages over nuclear medicine; and 4) 4D CT is a much less expensive procedure than other imaging modalities, which is an important consideration for clinical implementation.

In the current clinical practice of thoracic cancer radiotherapy, the differences in pulmonary ventilation and/or perfusion are mostly not considered when generating treatment plans. All regions of the lung are usually deemed equal. Clinicians and researchers have proposed to advance normal lung sparing in thoracic cancer radiotherapy by introducing ventilation and/or perfusion imaging into radiotherapy treatment planning.^25^, ^26^ If clinically implemented, it could lead to reduced radiation toxicity to lung regions with high perfusion and/or ventilation while adequate dose coverage of tumors is maintained. Since 4D CT has become widely accepted in thoracic cancer radiation therapy, logistically it is an excellent choice for ventilation calculation for the purpose of sparing functional lung during radiation dose treatment planning. However, as the ventilation data derived from 4D CT are introduced in clinical usage, further proper validation studies are needed.

This paper presents the *in vivo* evaluation of the 4D CT‐based geometric method of ventilation calculation, also called the ΔV method, and compares it with other two methods, the Jacobian and the HU methods, using previously published XeCT‐based ventilation data from studies on sheep *in vivo*.^(15)^

## II. MATERIALS AND METHODS

### A. Image data

Appropriate animal ethics approval was obtained for imaging acquisition protocols from the University of Iowa Animal Care and Use Committee and the study adhered to NIH guidelines for animal experimentation. Four adult male sheep were anesthetized and under positive ventilation during the experiments. 4D CT and XeCT scans were acquired with animal in the supine position. 4D CT images were reconstructed at different respiration phases. Zero% and 100% of the near end of expiration and near end of inspiration phases were used to calculate ventilation. XeCT scans were acquired with 12 axial slices over 45 breaths under respiratory gating at near end expiration. The XeCT based estimated regional lung ventilation was computed using Pulmonary Analysis Software Suite 11.0 by finding the constant of the exponential rise of the density from xenon gas wash‐in over multiple breaths. After resample, all images are with voxel dimension $1\times 1\times 1\;{\text{mm}}^{3}$. More details of the image acquisition protocols can be found in the previous publication.^(15)^

### B. Deformable image registration (DIR)

The Diffeomorphic Morphons (DM) method is based on matching edges and lines.^(27)^ The morphon iteratively deforms a moving image into a target image by morphing the former. The process can be divided into three parts: estimation of displacement, accumulation of the deformation field, and deformation.

Estimation of displacement needed to deform the moving image into the target image is based on quadrature phase difference.^(27)^ The accumulation of the deformation field uses the estimate of the displacement to update the deformation field. This is a two‐step process. The first step is to update the deformation field and to regularize the estimates of the accumulated field in order to fit the observed deformation to a deformation model. The second step is to morph the moving image to the target image according to the accumulated deformation field. These two steps are done iteratively as long as the displacement estimates are larger than a specified minimum displacement indicating acceptable convergence.

The quadrature phase difference method is used to estimate local displacement between two images. The advantage of this method over others, such as those based on gradient and polynomial expansion, is its invariance to image intensity and weak gradients.^(28)^ Quadrature phase is a measure of local structure. Edges between bright and dark areas, dark lines, lines on dark background, and bright patches, all have different phases. The transition from one phase to another is continuous. Therefore, the difference in local phase between the moving and target images is a good measure of how much the moving image has to move to fit the target image. The local displacement is a function of the local phase along its associated direction. To estimate the local displacement a least squares estimator is used as follows:

(1) $$\underset{v}{\text{min}}\sum_{i}\left[w_{i}{\left({\hat{n}}_{i}^{T}v-v_{i}\right)}^{2}\right],$$

where *v* is the displacement field estimate, $v_{i}$ is the displacement field associated with direction i, $w_{i}$ is a measure of certainty derived from the magnitude of the phase difference, and ${\hat{n}}_{i}$ is the unit normal vector for the i direction.

The displacement field of the current iteration is given by Eq. (1), which is used to interpolate a deformed version of the moving image, which is deformed based on the accumulated field and then compared to the target image in order to estimate a displacement field for the current iteration. The updated field (${d^{\prime }}_{a}$) is formed by combining the accumulated field ($d_{a}$) and the displacement field ($d_{k}$) from the current iteration.

(2) $$d_{a}^{'}=\frac{d_{a}c_{a}+(d_{a}+d_{k})c_{k}}{c_{a}+c_{k}},$$

where $c_{a}$ and $c_{k}$ are the uncertainties in the accumulated field and the current field, respectively. After acquiring the updated field in Eq. (2), as well as the uncertainty from the field, the following equation is used to determine the accumulated displacement field:

(3) $${\vec{d}}^{i}={\vec{d}}^{i-1}+\frac{c_{U}d_{a}^{'}}{c^{i-1}+c_{U}},$$

where $c_{U}$ is the uncertainty in the updated field.

The morphons method is optimized to become diffeomorphic. As a result, Eq. (3) becomes:

(4) $${\vec{d}}^{i}={\vec{d}}^{i-1}+\text{exp}\left(\frac{c_{U}d_{a}^{'}}{c^{i-1}+c_{U}}\right)$$

The deformation field produced by the DM is smoothed and iteratively used to transform the moving image and register it on to the target image. Smoothing is achieved using Gaussian regularization of the displacement field thereby reducing the influence of extreme values in a deformation field.

DM and diffeomorphic demons (DD)^(29)^ DIR methods were evaluated using the 4D CT data of the sheep with manually delineated landmarks in the end‐expiration and end‐inspiration phases in this study. The deformation transformations were used to map the landmarks from the expiration phase to the inspiration phase. The mapped landmarks were then compared with the manually delineated landmarks in the inspiration phase and the difference was defined as the target registration error (TRE). There were 220 landmark pairs in each case. The registrations were performed with the multiscale feature.^(30)^ Four levels of resolution were used in the registration, namely, 64×64, 128×128, 256×256, and 512×512. They were validated previously with average ± S.D. TRE (end‐expiration to end‐inspiration) was equal to 1.4±0.6 mm for DM and 1.4±0.7 mm for DD.^(30)^

Based on the evaluation results alluded to above, the DM DIR method was selected for the ventilation calculation in this study. The DM DIR computer program was developed by a group in the Netherlands.^(27)^ It runs within the MATLAB platform (MathWorks, Natick, MA). Each registration took about 3 hrs on a personal computer using a 3 GHz CPU. The deformation transformations from the 4D CT data were used for ventilation matrices calculation.

### C. Ventilation calculation

#### C.1 ΔV ventilation calculation algorithm

This study used the ΔV ventilation calculation algorithm^14^, ^31^ to derive ventilation from 4D CT data for four sheep. In the expiration phase of a 4D CT dataset, each voxel is a cuboid defined by eight vertices. In the inspiration phase, this cuboid is changed into a 12‐face polyhedron which is still comprised of the corresponding eight vertices. Any hexahedron or 12‐face polyhedron can be divided into six tetrahedrons. The volumes of the cuboid and the 12‐face polyhedron (deformed cuboid) are the sums of the volumes of their six corresponding tetrahedrons. In the inspiration phase, the DIR algorithm calculated the corresponding locations of the eight vertices that define the cuboid in the expiration phase. The volumes of each cuboid and the corresponding deformed cuboid are calculated using the corresponding vertices of each respective polyhedron.

The fundamental volume calculation is based on the volume calculation for each tetrahedron. The coordinates of the four vertices of a tetrahedron are used to determine its volume:

(5) V=(b−a)·[(c−a)×(d−a)]/6

where *a*, *b*, *c, d* are the vertices’ coordinates expressed as vectors. The volumes of the six tetrahedrons are summed up to generate the volume of the given polyhedron. The ventilation distribution was calculated as the distribution of,

(6) $$P=\Delta V/V_{ex}$$

where *ΔV* is the volume change between expiration and inspiration, and $V_{\text{ex}}$ is the initial volume for all voxels at expiration, which is constant across the whole exhale image.^(11)^

#### C.2 Jacobian algorithm

The Jacobian is a mathematical method where volume change is estimated using the first derivative of the deformation field.^13^, ^24^, ^32^, ^33^ Local volume change of the lung is calculated using the Jacobian of the transformation that maps the end expiration phase of 4D CT image to the end inspiration phase. Consider a vector displacement D(x,y,z) that transforms a voxel from its end expiration image to its corresponding location in the end inspiration image. The Jacobian operator J of this transformation is given by,

(7) $$\begin{array}{l} J=\text{det}\left[I+\left(\begin{array}{lll} \frac{\partial D_{x}(x,y,z)}{\partial x} & \frac{\partial D_{x}(x,y,z)}{\partial y} & \frac{\partial D_{x}(x,y,z)}{\partial z}\\ \frac{\partial D_{y}(x,y,z)}{\partial x} & \frac{\partial D_{y}(x,y,z)}{\partial y} & \frac{\partial D_{y}(x,y,z)}{\partial z}\\ \frac{\partial D_{z}(x,y,z)}{\partial x} & \frac{\partial D_{z}(x,y,z)}{\partial y} & \frac{\partial D_{z}(x,y,z)}{\partial z} \end{array}\right)\right],\\ F_{air}=-\frac{HU}{1000} \end{array}$$

where *I* is the identity matrix, $D_{x}$ *(x,y,z)* is the x component of D(x,y,z), $D_{y}$ *(x,y,z)* is the y component of D(x,y,z), and $D_{z}$ *(x,y,z)* is the z component of D(x,y,z). The determinant of the Jacobian is calculated at each voxel position according to Eq. (7). If the determinant of the Jacobian is unity, then no expansion or contraction of the voxel occurs. If the determinant is greater than one, there is local voxel (lung tissue) expansion; if less than one, there is local voxel contraction.

#### C.3 Hounsfield units (HU) method

The HU method uses deformable image registration to correlate voxels from the expiration image set to the anatomically corresponding voxels in the inspiration image. The change in density for each voxel is calculated by direct comparison of HUs.^11^, ^12^ The fraction of air in a CT lung volume may be estimated as,

(8) $$F_{air}=-\frac{HU}{1000}$$

where *HU* represents the average Hounsfield unit value within the volume. Let $F_{\text{exh}}$ be the fraction of air in the exhale CT volume, and $F_{\text{in}}$ the fraction of air in the inhale CT volume, then the change in volume is given by:

(9) $$\frac{\Delta V}{V_{ex}}=\frac{F_{in}-F_{\text{ex}h}}{F_{exh}(1-F_{in})}$$

Substitute Eq. (8) into Eq. (9), we get:

(10) $$\frac{\Delta V}{V_{ex}}=1000\frac{\left(HU_{in}-HU_{exh}\right)}{HU_{exh}(1000+HU_{in})}$$

#### C.4 Ventilation comparison

Due to the limits in dynamic scans, the volumes of the ventilation distributions by Xe‐enhanced CT imaging are usually limited to partial lungs. These partial lung distributions served as the masks in the ventilation distribution calculations with the 4D CT datasets. Thus the volumes with calculated ventilation matched with the XeCT data and the calculated ventilation distributions can be directly compared with the Xe ventilation images.

Image smoothing techniques are often used in image processing to reduce noise^(34)^ and an averaging filter is a simple smoothing technique.^(35)^ Different averaging filter sizes were applied to the 4D CT ventilation distributions before the comparison with the Xe ventilation distributions in order to reduce registration errors; these were 9×9×9, 19×19×19, and $29\times 29\times 29\;{\text{mm}}^{3}$ filters. Blood vessels and other high‐density regions were excluded in the ventilation calculation by all three methods. The ventilation value of the central voxel was the average value inside the box defined by the averaging filter. When the averaging filter mask was at the edge of the calculated ventilation region or where some ventilation values were not available due to the presence of blood vessels, the actual number of valid voxels inside the filter mask was used for the averaging calculation.

Because of the different metrics between the two ventilation imaging modalities, namely density difference for the XeCT versus volume change for the ΔV method, the two ventilation distributions were converted to the relative percentile distribution^(32)^ first and then the Dice similarity coefficient (DSC)^(36)^ was applied to calculate the similarity between the two ventilation volumes. When volumes A and B are compared, DSC is calculated as,

(11) $$DSC(A,B)=\frac{2\times \left|A\cap B\right|}{\left|A\right|+\left|B\right|}$$

The values of DSC index range between 1.0 and 0.0. ADSC of 1.0 indicates a complete similarity of the two volumes whereas a DSC of 0.0 indicates no similarity at all, with intermediate values describing proportional amounts of similarity. Similar to the cumulative dose‐volume histogram, if a certain percentage of lung volume is covered by no more than a certain ventilation value, this ventilation value is associated with the corresponding percentage value of the lung volume in the percentile distribution. The two aligned ventilation datasets were compared for similarity for the higher 30% and 50% of ventilation volumes (or 70 and 50 percentile).

Another way to compare the ventilation distributions is to calculate the Spearman correlation coefficient (SCC) between the ventilation data sets.^(17)^ The voxel‐wise correlation was calculated between the distribution by XeCT and the other three 4D CT methods for all four cases. The absolute ventilation distributions without conversion to percentile ventilation distributions were used for the SCC calculations.

## III. RESULTS

The average TRE with one standard deviation (1 SD) in the lungs for the four cases was 1.9±1.5 mm for DM and 2.0±1.6 mm for DD. The majority of the errors were within 3 mm, which is comparable to other studies^15^, ^37^ and consistent with our previous study.^(30)^ Based on the evaluation results, DM DIR was selected for the ventilation calculations in this study.

**Figure 1:**
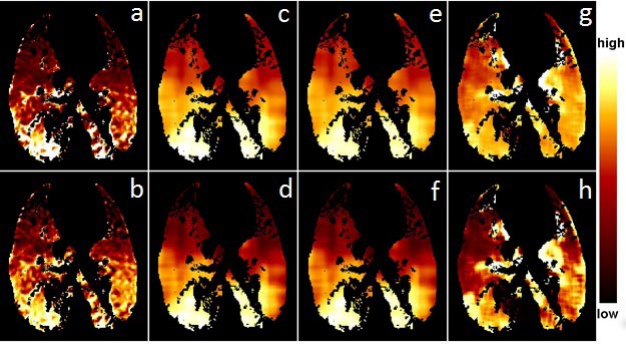
Ventilation distribution comparison between the XeCT and ΔV methods for best case: (a) absolute ventilation by Xe, (b) percentile distribution by Xe, (c) absolute ventilation by ΔV, (d) percentile distribution by AV, (e) absolute ventilation by Jacobian, (f) percentile distribution by Jacobian, (g) absolute ventilation by HU and (h) percentile distribution by HU. The averaging filter sizes used in this figure were 29×29×29 mm ^3^ for the ΔV and Jacobian methods and 9×9×9 mm ^3^ for the HU method.

**Figure 2:**
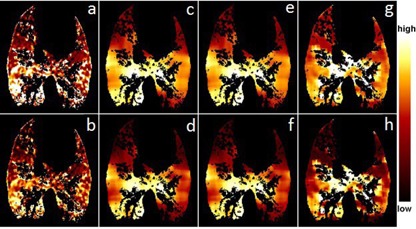
Ventilation distribution comparison between the XeCT and ΔV methods for the worst case: (a) absolute ventilation by Xe, (b) percentile distribution by Xe, (c) absolute ventilation by ΔV, (d) percentile distribution by ΔV, (e) absolute ventilation by Jacobian, (f) percentile distribution by Jacobian, (g) absolute ventilation by HU and (h) percentile distribution by HU. The averaging filter sizes used in this figure were 29×29×29 mm ^3^ for the ΔV and Jacobian methods and 9×9×9 mm ^3^ for the HU method.

The average Spearman coefficient *r* with 1 SD was 0.44±0.13 (range 0.29–0.61) between the XeCT and ΔV ventilation distributions over the four cases (sheep). It was 0.45±0.13 (range 0.31–0.61) between the XeCT and the Jacobian ventilation distributions, and 0.30±0.10 (range 0.17–0.42) between the XeCT and the HU ventilation distributions. Based on the Spearman coefficient, Figs. 1 and 2 show the ventilation distributions for the best (r=0.61) and worst (r=0.29) cases, respectively, between XeCT, and XXDV, Jacobian, and HU methods. The qualitative agreements are patent by visual inspection.

The average DSC value for the upper 50% ventilation volumes between the XeCT and ΔV ventilation distributions was 0.67±0.05 (range 0.61–0.74), while for the upper 70% ventilation volumes, it was 0.81±0.03 (range 0.78–0.84). The average DSC results between XeCT and Jacobian ventilation distributions were the same (the individual values were different), while between XeCT and HU they were 0.63±0.05 for the upper 50% ventilation volumes (range 0.58–0.68) and 0.79±0.03 for the upper 70% ventilation volumes (range 0.74–0.81). The similarity of the lower 30% ventilation volumes was 0.55±0.07 between the XeCT and ΔV and 0.52±0.08 between the XeCT and HU, respectively.

**Figure 3:**
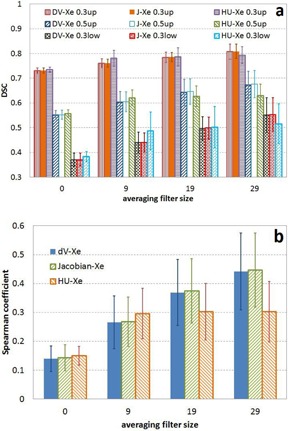
Mean Dice similarity coefficient (a) vs. averaging filter size and (b) mean Spearman coefficient vs. averaging filter size over the four cases between XeCT ventilation distribution and ventilation distributions calculated using 4D CT.

It was found that the similarity between the ventilations calculated using 4D CT and XeCT depends on the averaging filter size applied to the 4D CT ventilation data. Figure 3(a) shows the mean Dice similarity coefficient versus averaging filter size and Fig. 3(b) the mean Spearman coefficient versus averaging filter size for the four cases. The dependence of the HU method on filter size was flat with the size larger than 9×9×9 mm ^3^. At the filter size of 9×9×9 mm ^3^ and smaller, HU method demonstrated better similarity to the ventilation distribution from XeCT than the other two methods, while with the larger filter size, the other two methods showed better similarity than HU method.

**Figure 4:**
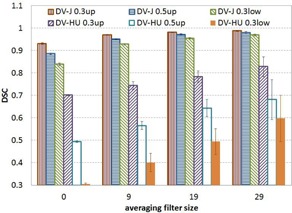
Mean dice similarity coefficient vs. averaging filter size and ventilation volume between ΔV ventilation and those calculated by Jacobian and HU methods.

Figure 4 shows the mean DSC versus averaging filter size and ventilation volume between the ventilation calculated using ΔV and the ones calculated using the Jacobian and HU methods. DSC was high between the XXDV and Jacobian methods, while DSC was strongly dependent on ventilation volume and averaging filter size between the ΔV and HU methods; more smoothing (larger filter size) resulted in better similarity.

## IV. DISCUSSION

No matter how accurate the deformable image registration algorithm is, registration errors are inevitable due to the noise inherent in CT images.^(38)^ The registration errors in DIR are the reason for the similarity dependence on averaging filter size. The averaging is applied to smooth the ventilation distributions. The ventilation difference introduced by the registration error is reduced by smoothing, and the resulting ventilation distribution is thus closer to that obtained with XeCT.

The other major source of errors in DIR is artifacts in CT images. The sheep 4D CT data used in this study did not show obvious artifacts because they were mechanically ventilated and therefore breathed regularly. For clinical human 4D CT, motion artifacts exist when irregular motion is involved, in which mushroom artifacts due to the irregular motion of the large surface of the diaphragm are most obvious.^(39)^ Such artifacts introduce DIR errors and images with such artifacts should not be used in ventilation calculations.^(40)^

The HU method needs less smoothing compared to the other two methods because during the ventilation calculation it excludes voxels in which large density difference occurs after registration. The registration errors often yield nonperfect matches between the two phases of a 4D CT dataset, such as chest wall‐lung tissue and blood vessels‐lung tissue interfaces, which could introduce large density differences at such interfaces.^(14)^ These edge artifacts are easily removed during the ventilation calculation using the HU method by checking if any of the involved two sets of CT data has a higher HU number than normal lung tissue for any given voxel. The ventilation values for the artifact voxels were set to the values of the closest valid voxels. Because of the removal of the large errors during the ventilation calculation, HU method does not need much smoothing of the resulted ventilation distribution. For the other two methods, the deformation transformation from the registration is used to calculate the volume change, and the volume change errors introduced by the registration errors are not easily removed in the ventilation calculation. Additional smoothing of the ventilation distribution is an effective way to reduce the ventilation errors.

Both ΔV and Jacobian methods calculate ventilation based on the local volume change. One performs a direct geometric calculation and the other approximates volume change using the Jacobian determinant of a transformation. The volume change calculations are performed based on the same deformation transformation in each case. Thus the ventilation distributions generated by these two methods are similar to each other. HU method calculates ventilation based on the local HU change. Although the same deformation transformation is used, different target properties from the transformation are used in the calculations. The resulted ventilation distributions could be different due to registration errors and thus can be reduced by smoothing, which is seen in Fig. 4.

Because the accuracy of the ventilation distribution generated from 4D CT data and deformable image registration depends on the accuracy of the registration, which in turn depends on the registration algorithm and the quality of the 4D CT data, high‐quality 4D CT data are essential for producing accurate ventilation calculation^(38)^ — registration algorithms need to be validated for such applications.^(41)^

Validation of the ΔV‐4D CT ventilation calculation method is important because 4D CT‐based techniques are finding medical applications given that these scans are faster and do not require injection of radioactive materials or contrast agents. For example, 4D CT‐based ventilation has been used in radiation therapy treatment planning to avoid functional lung and to estimate loss of lung function or normal tissue toxicity after radiotherapy in a dose‐dependent manner.^40^, ^42^, ^43^, ^44^, ^45^ Additional medical applications, such as in diagnostic imaging, may be possible.

## V. CONCLUSIONS

Ventilation distributions calculated using 4D CT and deformable image registration were compared to the ventilation distribution derived from Xe‐enhanced CT data, which was considered the gold standard, for four sheep. The 4D CT‐based ventilation distributions agreed with the ones from XeCT reasonably well. Ventilation differences introduced by registration errors can be reduced by smoothing. This evaluation study and previous reports^15^, ^18^ support the use of ventilation calculated using 4D CT in clinical applications such as radiation treatment planning,^12^, ^23^, ^24^, ^40^, ^42^, ^43^, ^44^, ^45^ pulmonary function,^15^, ^18^ and lung disease.^(22)^

## COPYRIGHT

This work is licensed under a Creative Commons Attribution 4.0 International License.
